# New measurement criteria for studying alcohol drinking and relapse in rodents

**DOI:** 10.1186/2193-9616-1-13

**Published:** 2013-11-01

**Authors:** Lilian Villarín Pildaín, Valentina Vengeliene, Franziska Matthäus

**Affiliations:** Max Planck Institute for Molecular Genetics, Berlin, Germany; Central Institute for Mental Health, Institute of Psychopharmacology, University of Heidelberg, Faculty of Medicine, Mannheim, Germany; Department of Neurobiology and Biophysics, Faculty of Natural Sciences, Vilnius University, Vilnius, Lithuania; Center for Modeling and Simulation in the Biosciences (BIOMS), University of Heidelberg, Heidelberg, Germany

**Keywords:** Alcohol deprivation effect, Relapse, Drinking patterns, Animal model of addiction, Four-bottle paradigm

## Abstract

**Purpose:**

Relapse to alcohol use is considered as one of the central features distinguishing dependence from controlled alcohol consumption. Relapse-like drinking in rodents is a transient episode of heavy drinking that follows a period of abstinence. This behaviour is called the alcohol deprivation effect (ADE). Not all animals develop behavioural changes that resemble relapse-like drinking behaviour. The purpose of our study was to develop a generalized quantitative criterion by which animals could be separated into two groups depending on their behaviour during a relapse-like situation (ADE vs. no-ADE).

**Methods:**

An automated drinkometer system was used for data collection. This system measures fluid consumption by means of high-precision sensors attached to the drinking bottles in the home cage of the rat. We used a four bottle free choice paradigm with water 5, 10, and 20% ethanol solutions. For data analysis we developed a new measure of alcohol intake that quantifies net alcohol intake in relation to net consumption of water. This new measure is called water-penalized net ethanol intake.

**Results:**

The new measure is more robust than commonly used measurements, such as alcohol preference and intake. It allows the comparison of alcohol intake between different groups of animals and different setups using an arbitrary number of bottles. Based on this new measure we developed a method to automatically select the threshold for the presence of ADE in individual animals.

**Conclusions:**

Separating animals by their behavior during relapse-like situation could be used as one of the criteria for identification of alcohol addicted and non-addicted rats. A classification into presenting ADE or not is also essential to test the effectiveness of newly developed therapeutic drugs.

## Background

Even though a great number of people consume alcohol, only a few of them develop dependence. Depending on various modulating factors, such as genetic predisposition, provocative environmental experiences, pharmacological history and social context, controllable alcohol intake could become compulsive (Spanagel 2009). It is believed that dependence appears only in certain individuals because of their specific, innate or acquired, qualities, which define response to the drug and the likeliness in developing dependence (Piazza and Le Moal 1996).

If an individual becomes addicted, relapse to alcohol use is considered as one of the central features distinguishing dependence from controlled alcohol consumption. Relapse episodes occurring after a long-lasting interruption in drug consumption, i.e. long after withdrawal syndromes have past, reflect the failure to control alcohol consumption by the addicted individual and it is the major problem in the treatment of alcoholism in humans. In our previous studies we used an animal model of alcoholism described in detail by Spanagel and Hölter (1999). This model is focused on the relapse-like behaviour. Modeling the entire spectrum of a complex human mental disorder such as addiction in animals is not possible due to its complexity. But we can translate anthropomorphic terminology into objectively and behaviourally measurable parameters and thus model at least some key criteria of the disorder. With regards to relapse behaviour, this is a straight forward endeavour as a relapse is defined as the recurrence of a past condition, namely excessive and uncontrolled drinking after a phase of abstinence. The alcohol deprivation model provides excellent face validity to relapse behaviour seen in alcoholics (Vengeliene et al. 2014). However, not all animals exhibit an alcohol deprivation effect (ADE) and only a smaller percentage develop compulsive drinking.

Our goal was to develop a generalized criterion enabling us to separate the rats that show relapse-like behaviour (ADE) from the rats that do not show such behaviour (no ADE). This kind of classification is essential for research on addiction, for instance when testing the effectiveness of therapeutic approaches as exemplified recently with a DSM-IV based animal model of cocaine addiction (Deroche-Gamonet et al. 2004; Cannella et al. 2013). Our here presented approach encompasses data-driven mathematical modeling and statistical analysis, and the development of a new alcohol intake measure and classification method.

Our new alcohol intake measure relates the amount of consumed net alcohol to the uptake of water (either pure or in alcoholic solution). We show that this measure has certain advantages in comparison to other measures, like net EtOH intake or preference. Based on this new intake measure, we develop a framework to identify ADE/non-ADE in individual animals.

One of the few approaches to mathematically model relapse-like alcohol consumption in rats was developed by Sinclair et al. (Sinclair et al. 1973; Sinclair and Li 1989). Here, increased alcohol intake following a deprivation phase was fitted to an exponential decay model, which accounts for over 99% of the variance from the mean alcohol intake. These results were obtained under a two-bottle free choice paradigm (water and either 7% or 10% EtOH). Concurrent access to four bottles (water and three different alcohol solutions) were used in the present study. In addition, we used a drinkometer system, a novel device for high time resolution measurement of fluid intake (Vengeliene et al. 2013). The availability of high time-resolution drinking measurements for the four-bottle setup requires the adaptation of the ADE criterion described by Sinclair et al. (Sinclair et al. 1973; Sinclair and Li 1989), since the net ethanol intake or solution preference does not meaningfully make use of all the acquired information. With our new measure of alcohol intake and the individual classification of animals into ADE or non-ADE, the increase in alcohol intake during relapse can be fitted to the model of Sinclair. Our model parameters are in perfect agreement with the estimation of Sinclair. Alternative measures of alcohol intake or ADE classification procedures yield very poor agreement with the model.

The compulsiveness of alcohol consumption during the relapse-like drinking can be measured using a taste adulteration test. In this test the taste of alcohol solutions is altered with the bitter quinine (Wolffgramm and Heyne 1995; Spanagel et al. 1996). A rat is expected to naturally choose less aversive fluid as a drinking source Brasser et al. (2012). Those animals that exhibit an ADE despite alcohol taste adulteration with the quinine are classified as compulsive animals. The quinine test was used for verification of the new ADE criterion.

Summarizing, our new measure of alcohol intake is more robust than other measures of alcohol intake, allows ADE classification of individual animals, and uncovers important features of alcohol drinking patterns under the protocol of free-choice and arbitrary number of alcohol bottles.

## Methods

### Animals

Twenty-nine two-month-old male Wistar rats (from our own breeding colony at the CIMH, Mannheim, Germany) were used. All animals were housed individually in standard rat cages (Type-III; Ehret, Emmendingen, Germany) under a 12/12-hour artificial light–dark cycle (lights on at 7:00 a.m.). Room temperature was kept constant (temperature: 22 ± 1 °C, humidity: 55 ± 5%). Standard laboratory rat food (Ssniff, Soest, Germany) and tap water were provided *ad libitum* throughout the experimental period. Body weights were measured weekly. All experimental procedures were approved by the Committee on Animal Care and Use, and carried out in accordance with the local Animal Welfare Act and the European Communities Council Directive of 24 November 1986 (86/609/EEC).

### Drinkometer system

The drinkometer system has been developed together with TSE Systems (Bad Homburg, Germany). It enables continuous long-term monitoring of liquid consumption by amount and time in a standard rat home cage (Eurostandard Type III). The system is equipped with four drinking stations to allow liquid choice. The drinking station consists of a glass vessel containing the liquid and a high precision sensor for weighing the amount of liquid removed from the glass vessel (Vengeliene et al. 2013). Monitoring of all drinking stations is carried out by a computer. The system features ultra-high resolution-down to 0.01 g. The whole system is mounted to a custom-made free-swinging steel frame in order to avoid any environmental disturbances. The drinkometer system measures the weight of a vessel in 200 ms steps and saves it in 1 s steps. The normal sampling can be set with minimum 1 min intervals. For the present study, sampling was performed at 5 min intervals.

### Long-term alcohol consumption with repeated deprivation phases

After two weeks of habituation to the animal room, rats (n = 29) were given ad libitum access to tap water and 5%, 10% and 20% ethanol solutions (v/v). The first two-week deprivation period was introduced after eight weeks of continuous alcohol availability (baseline drinking). After this deprivation period, rats were given access to alcohol again. This access was further interrupted repeatedly with deprivation periods in a random manner (i.e., the duration of following drinking and deprivation phases was irregular, i.e. approximately 4-5 weeks and 2-3 weeks, respectively in order to prevent adaptive behavioural mechanisms). Baseline measurements of fluid intake every 5 min yielded long time series (20 days of 288 daily measurements). Post-abstinence ethanol consumption (ADE) was measured for one week. Our analysis was based on the data collected from the first, the third and the fifth cycle of repeated baseline/deprivation phases. During the 1^st^ after-deprivation (AD) phase, 0.05 g/l of quinine was added to the ethanol solutions for half of the animals. During the third phase, no animal received quinine. During the 5^th^ AD phase, the second half of animals received quinine-adulterated ethanol solutions. To validate the robustness of our results, a different group of rats (n = 22) received quinine-adulterated ethanol solutions during the 9^th^ AD phase.

### Statistical tools

The following statistical tools were used to model the increase in alcohol intake with the aim to identify the ADE. We compared two groups of animals: one group which received quinine-adulterated alcohol solutions after a deprivation phase, and a control group that received quinine-free solutions.

A) We assume that the increase in alcohol intake during the ADE in the control group follows a normal distribution, for which we obtained maximum likelihood estimators of the parameters (mean (μ) and variance (σ^2^)). For both groups we then computed the likelihood of the increase in alcohol intake (here x) to be drawn from the fitted normal distribution:
  This provided the likelihood that the increase of alcohol intake of an individual rat (from either the control or the quinine group) is well represented by (and does not deviate much from) this normal distribution.
B) Furthermore, we also needed to know if a subgroup of all animals (comprising only control, or only quinine animals, or a mixed selection) was well represented by the fitted normal distribution. This involved the comparison of distributions, for which we used the Kolmogorov-Smirnov test (*KS-test*). Shortly, this test explores whether a sample {*x*_1_,…,*x*_*N*_} comes from a given continuous cumulative distribution *F*. For this, one computes the empirical cumulative distribution of the sample *F*_*N*_ and the test statistic:
  Under the null-hypothesis that the sample {*x*_1_,…,*x*_*N*_} comes from the cumulative distribution *F*, the statistic *KS*_*stat*_ is Kolmogorov distributed (Massey 1951), and we find p-values for the Kolmogorov distribution. The assumption from A) that that the increase in alcohol intake during the ADE in the control group follows a normal distribution was supported by high p-values of the KS-test (p = 0.45), thus the hypothesis of normality of the distribution could not be rejected.
C) The Kolmogorov-Smirnov test was then used to define a threshold for classifying animals into presenting ADE or not. The threshold θ was chosen such that ADE classified animals under this threshold maximize the Kolmogorov-Smirnov p-value for the hypothesized normal distribution. In other words, θ was selected to obtain the group of animals (regardless of the condition) which provided the best fit to the model under the hypothesis that ADE is a phenomenon independent of (quinine) taste-adulteration.
D) We then still had to test whether this classification (into ADE or no ADE) was affected by quinine or differed between different deprivation cycles. For comparing group differences or similarities in classification we used contingency tables. Contingency tables are used to test whether two (or more) classifications of the same group (here: quinine group / control group, and ADE / no ADE) are independent from each other (i.e. whether the probability of exhibiting an ADE depends on the presented solution). In our case the small sample size led to small entries of the table which made the use of Pearson χ^2^ test for contingency tables not applicable. Instead we used the Fisher’s exact test (Agresti 1992), which was specifically developed to deal with small sample sizes. Fisher’s exact test computes how extreme the observed table is, under the null hypothesis of independence and fixed marginals. It computes all possible tables with same row and columns totals (as the observed table) and its probabilities under the null hypothesis of independence, in which case the sets of values in the table follow the hypergeometric distribution.

## Results

### Alcohol intake measures

When only a single ethanol solution is available, its effect can be easily analyzed and compared across individuals. However, less concentrated alcohol solution takes longer time to ingest and therefore has a different effect on the brain (15 ml of pure alcohol corresponds to 300 ml of 5% solution but only to 75 ml of 20% solution). The amount of accompanying water, as well as the time required to drink the amount of net alcohol varies greatly. This should be taken into account in a multiple-concentration approach, i.e. in free choice drinking procedure from multiple bottles water vs. different concentrated ethanol solutions with ≥3 bottles. We propose therefore a new measure which we call water-penalized net ethanol (EtOH) intake. Given the amount drank of each solution EtOH_*p*_*with p* = {0.05; 0.1; 0.2}, the water-penalized net EtOH intake is calculated as

This equation quantifies the fraction of the net alcohol intake against H_2_O provided through each solution. It penalizes drinkers that drink simultaneously a large amount of water and gives a stronger effect to alcohol consumed from higher concentrations.

In the following, we present alcohol intake measures that are commonly used for two-bottle setups, and describe their advantages and disadvantages when applying them to data from a multiple-bottle setup (≥3 bottles):

One possibility to measure alcohol intake is by ethanol preference. Given the amount of drank H_2_O and each solution EtOH_*p*_, *p =* {0.05, 0.1, 0.2}, the ethanol preference is calculated as

This equations quantifies the ethanol preference, but not the fraction of alcohol intake each solution provided.

Another possibility to quantify alcohol intake is to compute the alcohol intake (ml per kg body weight): Given the consumed amount of each solution EtOH_*p*_, *p* = {0.05; 0.1; 0.2}, the net EtOH intake is calculated as

This equation gives the fraction of the net alcohol intake (ml) per kg body weight provided through each solution. Note that the proportional measure (g/kg) can be obtained multiplying this equation by the density of EtOH (0.789 g/ml). This measure, however, does not take into account how much water was simultaneously drunk and can be furthermore affected by sudden gain/loss of body weight.

**Figure 1:**
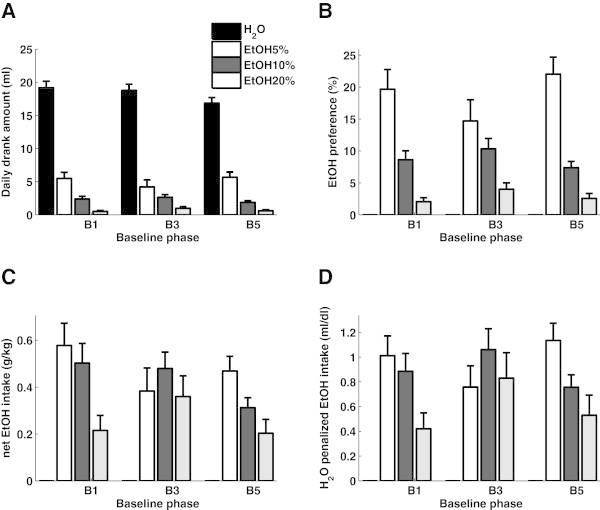
**Graphic representation of standard measures (A,B,C) for alcohol intake and the H**_**2**_ **O penalized net EtOH (D) shown for different baseline phases (B1, B3, and B5).** Consumed amount **(A)** and preference **(B)** give a general overview of the intake but do not provide a measure of the net ethanol consumption. The classical measure of ethanol intake **(C)** is modified to include information on the additional consumption of water (water-penalized net EtOH intake **(D)**).

**Figure 2:**
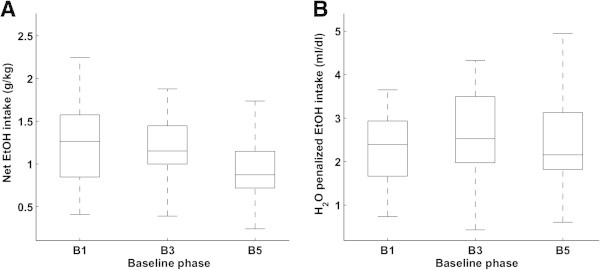
**Ethanol intake in g** **per kg** **body weight (A) is the standard intake measure.** However, it tends to decrease with subsequent baseline/deprivation phases for the same group of animals (B1, B3 and B5). This decrease is affected by the growth of the animals (weight increases), and by a reduction in water consumption. The H_2_O-penalized net EtOH intake **(B)** measures the net ethanol consumption per volume of water, and remains constant throughout phases. Each box-plot represents the distribution of the intake during each baseline. The box lower, middle and upper horizontal lines represent lower, median and upper quartiles. Horizontal lower and upper lines outside each box represent minimum and maximum values of the represented data. Outliers are not displayed.

The ethanol preference is proportional to the daily consumed amount of each solution, and for baseline drinking usually decreases with increasing alcohol concentrations (see data from baseline 1, 3, and 5 shown in Figure 1A and B). The preference measure gives a general overview over the drinking behavior, but provides no information on the net alcohol intake. For these measures the drinking profile differs from the ethanol preference profile (Figure 1C and D). While the intake profiles described by ethanol intake in g/kg body weight and water penalized net EtOH intake look very similar, the overall ethanol intake tends to decrease with subsequent baseline phases (Figure 2A), since the animals gain weight and reduce water consumption. The water-penalized net EtOH intake is stable throughout the consecutive baseline phases (Figure 2B).

### Identifying ADE in individual animals

With the proposed new alcohol intake measure it is also possible to identify if, after a deprivation phase, a given animal presented an ADE or not. Hereby the test is only applied to animals receiving quinine-adulterated alcohol solutions, because most Wistar rats present an ADE without adulteration of ethanol solutions with quinine (Sinclair and Senter 1967Sinclair et al. 1973; Spanagel and Hölter , 1999Spanagel et al. 1996). On the other hand, rats find the taste of quinine aversive. Therefore, quinine-adulteration results in an interruption/decrease in alcohol intake. An ADE and thus uncontrolled and compulsive drinking behavior is given if, after a period of abstinence, the rats increase their quinine-ethanol intake (with respect to baseline) to the same level as controls (Spanagel et al. 1996). In the following we describe a procedure that allows identifying an ADE in quinine containing ethanol solutions in individual animals.

To derive an ADE criterion we assume that on each day following deprivation, the average increase in water-penalized EtOH intake (*I*_*d*_) distributes normal with parameters μ_*d*_ and σ_*d*_^2^ for the days *d =* {0, 1, 2, …}. Furthermore, we know that during the first 2 AD days, the increase is most significant. Therefore, only these two days are used to determine the ADE. In summary, each rat *r* is characterized by the mean water-penalized net EtOH increase on the first two AD days:

 which distributes normal with parameters  and .

**Figure 3:**
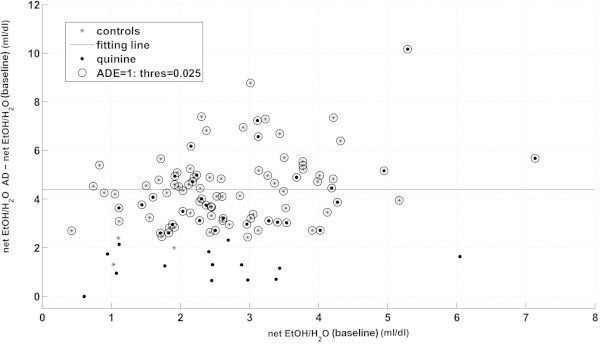
**ADE classification: Mean H**_**2**_ **O-penalized net EtOH increase of the first 2 AD days (y-axis) as a function of the H**_**2**_ **O-penalized EtOH intake during baseline drinking (x-axis).** No dependence of the increase in alcohol intake after deprivation on the alcohol intake during the preceding baseline can be observed, so the increase can be modeled as a normally distributed random variable. A normal distribution (whose mean is depicted as a solid line) is fitted to the controls, and the likelihood of all animals given the fitted parameters is computed. Animals presenting ADE are selected to have likelihood greater than a threshold value (circles).

Note that *I*^*r*^ can be modeled as a normally distributed random variable because the increase in consumed alcohol intake after a deprivation phase does not depend on the amount of consumed alcohol during the preceding baseline phase (Figure 3).

In the first step, a maximum likelihood estimator for the parameters μ_C_ and σ_C_^2^ (for the control group) is obtained, fitting {*I*^*rc*^}_*rc*  ∈  *controls*_ to the model. The next step is to compute the likelihood *L*(*I*^*r*^|*μ*_*C*_, *σ*_*C*_) of each animal (quinine and controls) to be represented by a normal distribution with the parameters *μ*_*C*_ and . The animals are classified into presenting ADE (1) or no ADE (0) by setting a threshold θ, and:

Since we assume that all animals presenting ADE have an increase in alcohol intake that distributes normally, the threshold θ is selected to maximize the likelihood of the *ADE*(*r,*θ)=1 group, given the fitted parameters for the control group. For this we systematically vary θ and compute for every value of θ the *p-value* of the Kolmogorov-Smirnov-test. This test inspects the H_0_ hypothesis, that {*I*^*r*^|*ADE*(*r*, *θ*) = 1} distributes normal with parameters *μ*_*C*_ and .

**Figure 4:**
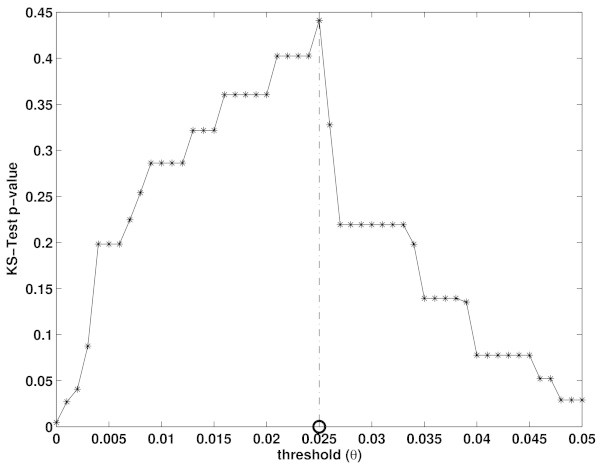
**A grid of threshold values (θ) for**  **is inspected (x-axis).** Animals having  are classified as presenting ADE, the rest as no-ADE. ADE classified animals are then tested through the Kolmogorov-Smirnov-test for equality of distributions, with  the hypothesized continuous distribution. Obtained *p-values* (y-axis) depend on the threshold θ. The threshold yielding the maximal *p-value* is selected (in our case, θ = 0.025).

The threshold was then selected to maximize the corresponding *p-value*. For our data set we obtained θ = 0.025. Figure 4 shows the *p-value* of the Kolmogorov-Smirnov-test as a function of the threshold θ.

### Classification results and validation

#### A) Comparing different sets of animals

**Figure 5:**
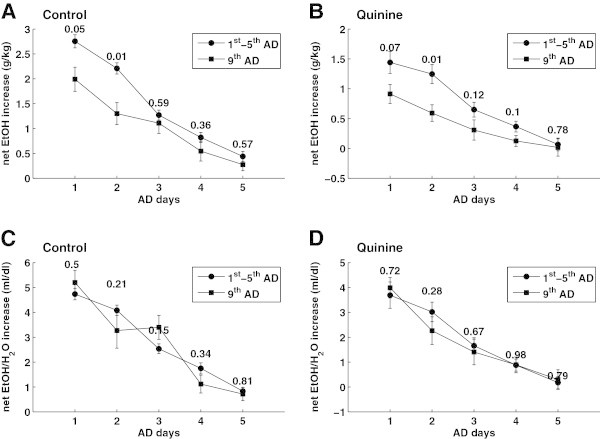
**Ethanol intake in g/kg body weight (A and B) differentiates between the increase in the first group of animals (1** ^**st**^ **-5** ^**th**^ **AD phases) and the second group (9** ^**th**^ **AD phase).** H_2_O penalized net EtOH intake **(C and D)**, however, shows a similar intake for both controls and quinine animals from both data sets. *P-values* are computed from a two-sample t-test.

To further validate the robustness of our intake measure we compared alcohol intake following a deprivation phase between two sets of animals. Data from 1^st^, 3^rd^, and 5^th^ ADE stem from one group of animals. For comparison we use data from a 9^th^ ADE of a different group of rats. We see that that intake in g/kg body weight differs considerably between the two groups (1^st^, 3^rd^, and 5^th^ versus 9^th^ ADE) (Figure 5A, B). The water penalized net EtOH intake, however, behaves similarly for both data sets (Figure 5C and D).

#### B) Dependence on deprivation cycle and quinine-adulteration

Furthermore, we tested if the distribution of animals presenting ADE and no-ADE was dependent on the deprivation cycle (1^st^ vs. 3^rd^ vs. 5^th^ cycle) or quinine-adulteration. For this analysis we used contingency tables and obtained p-values through Fisher’s exact test. This analysis yielded the following results: Around 95% control animals present ADE regardless of the deprivation cycle, which is corrobated by a high p-value (0.55) from Fisher’s exact test. For animals receiving quinine-solutions, the distribution of animals into presenting ADE or no-ADE in the first cycle was significantly different (p-value below 0.04) than in the advanced phases. In the first phase less than 43% of all animals presented an ADE. In later phases, the distribution into ADE or no-ADE is stable and no significant difference could be observed. Hence, around 80% of the animals present an ADE in the 3^rd^ and 5^th^ cycle. For details see Table 1.

**Table 1:** **ADE classification throughout different AD phases for controls (A) and quinine (B) animals**

**(A) Controls: Fisher’s** ***p-value*** **= 0.86**			
	**ADE = 0**	**ADE = 1**	**Row totals**
1^st^ AD	1	14	15
3^rd^ AD	1	28	29
5^th^ AD	1	13	14
9^th^ AD	0	7	7
Col. totals	3	62	65
**(B) Quinine: Fisher’s** ***p-value*** **= 0.07**			
	**ADE = 0**	**ADE = 1**	**Row totals**
1^st^ AD	8	6	14
5^th^ AD	3	12	15
9^th^ AD	3	11	14
Col. totals	14	29	43

The animals are classified into presenting ADE (1) or no ADE (0).

No significant difference in the distribution of classification between different phases or groups can be inferred (Fisher’s *p-value* = 0.86 (A) and 0.07 (B)). However, the low *p-value* in the quinine group (B) lead us to further tests: comparing 5^th^ and 9^th^ quinine AD: Fisher’s *p-value* = 1; comparing 1^st^ quinine AD versus 5^th^ and 9^th^ quinine AD: Fisher’s *p-value* = 0.0347.

#### C) Validation of classification and comparison with data from a 2-bottle paradigm

**Figure 6:**
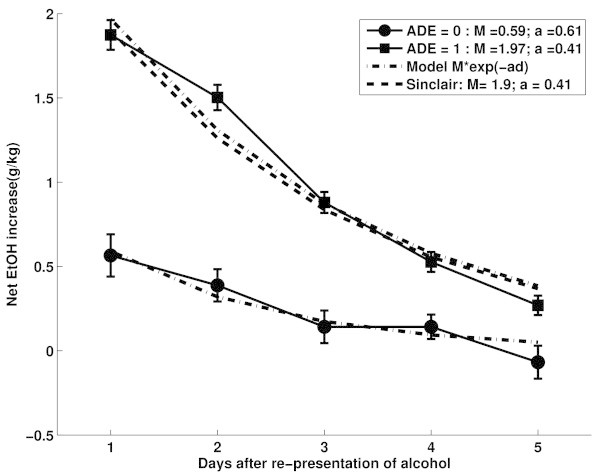
**Sinclair’s model fitted to ADE (1) and no-ADE (0) animals: the fitted parameters for ADE animals** ***M*** **= 1.97 and** ***a*** **= 0.41 are very similar to those reported by Sinclair in(Sinclair and Senter** 1967 **).**

In order to validate the obtained classification, we fitted our data to the model of AD-ethanol intake increase proposed in (Sinclair and Senter 1967; Sinclair 1971). The study was performed under the two-bottle choice protocol (H_2_O and EtOH_*p*_, with *p* = 0.10 commonly used), and based on home cage measurements. The model describes the decay of AD increase with successive AD days as an exponential, for net EtOH intake and preference. I.e. it models

with *I*_*AD*_*(d)* the increase in alcohol intake at day *d, A* the amount of consumed EtOH and P the preference, AD denoting after-deprivation and B referring to the baseline, as a function of the amount of days *d* = {0, 1, …} after re-presentation of alcohol following a deprivation phase:

where *d* = {0, 1, … } is the amount of days after representation of alcohol and *M* and *a* are the model parameters to be fitted to the data. Parameter values are given that account for 98% of the daily variation (*a* = 0.40; *M* = 1.9 g/kg and M = 22 for net EtOH increase and preference respectively). Figure 6 depicts the curve 1.9e^-0.4*d*^ (discontinuous (−−) line). In analogy to the approach of Sinclair *et al.* we computed the increased water-penalized EtOH intake of successive AD days with respect to baseline for ADE and non-ADE animals. Both groups were fitted to an exponential decay. The fitted parameters of the animals classified as presenting ADE (*M* = 1.97 g/kg and *a* = 0.41) agree very well with the values obtained by Sinclair *et al.* The fit for no-ADE classified animals, on the other hand, yields very different parameters (*M* = 0.59 and *a* = 0.61).

Figure 6 shows the data both groups, where dots depict daily mean increase and bars standard errors of non-ADE (circles) and ADE (squares) classified animals. Both groups were fitted and the model fits are depicted in Figure 6 with discontinuous (−.) lines. Observe the similarity between the Sinclair (−−) curve and the fitted model for ADE classified animals.

#### D) Robustness with respect to variations in the input space

To test whether the classification is robust with respect to changes in the set of animals included, we repeatedly removed 20 animals (over 20%), classified the remaining animals, and compared the result to the classification based on the entire sample. Based on 500 repetitions of this procedure we computed a misclassification rate per individual. The average misclassification rate was very low (2%) although we used less than 80% of all animals, which indicates high robustness of our classification method with respect to variations in sample size and composition.

## Discussion

In the present report we achieved two goals–we developed a generalized quantitative criterion enabling us to separate the rats that show relapse-like behaviour (ADE) from the rats that do not show such behaviour (no-ADE). Furthermore, we propose a new measure for alcohol consumption in a free choice home cage drinking paradigm with multiple bottles containing water vs. different alcohol solutions. This water-penalized net EtOH intake measure applied to a two-bottle free choice paradigm yields the same results as any other standard measure such as ethanol intake and preference; however, in a four-bottle paradigm it can uncover important features that are not discernible when standard measures are used.

The proposed measure penalizes the impact of alcohol in a free-choice protocol, depending which solution was consumed. Thus, 15 ml of pure ethanol in 300 ml water (which corresponds to a 5% v/v ethanol solution) has different pharmacokinetic and most likely pharmacodynamic effects than the same amount of ethanol in 75 ml water (which corresponds to a 20% v/v ethanol solution). The amount of accompanying water, as well as the time required to drink the amount of net alcohol varies greatly. This should be taken into account in a multiple-concentration approach, i.e. in free choice drinking procedures from multiple ethanol concentrations. The new measure furthermore does not suffer from many drawbacks of characteristic to other measures, such as: (i) a decreasing trend in alcohol consumption throughout deprivation phases. This is an artifact arising from the fact that animals reduce the net amount of alcohol, but accordingly decrease their water intake, so that the alcohol/water ratio is actually almost constant throughout phases. The new intake measure is, by construction, free from this artifact. (ii) The paradox of high-level alcohol drinkers not presenting ADE: high-level drinkers can not easily outperform their net alcohol intake after deprivation, and thus do not present ADE. However, by drinking less water, or by consuming stronger concentrated solutions, they can outperform their water-penalized EtOH intake, and can thus be regarded as presenting ADE. (iii) The new measure also normalizes the after-deprivation increase in alcohol intake for different phases and different sets of animals. This way we found that, independently of the net amount of alcohol consumed by the animal (which varies greatly from rat to rat and even for the same rat during different phases), there is a limit in the increase of ml of alcohol per dl of water a rat can achieve, probably related to metabolic mechanisms.

A further strength of the method is that it makes experimental designs with two solutions (water vs. alcohol) and four solutions (water vs. different alcohol concentrations) comparable. As an example we used the post-abstinence alcohol intake dynamics developed by Sinclair and Senter. Here, Sinclair et al. (Sinclair and Senter 1967; Sinclair 1971), using a 2-bottle setup, found an exponential decay of alcohol intake in the days following a deprivation phase. With our automatic ADE classification procedure and the water-penalized alcohol intake measure we obtained similar parameters, using the four-bottle setup, for the animals classified as presenting ADE. Thus, the drinking behavior of the ADE-presenting group showed the same dynamics in a 4-bottle setup as that shown by Sinclair and Senter for a 2-bottle setup. No-ADE animals, however, were described by an exponential decay with very different parameters. Thus, the drinking behavior of the ADE group showed the same dynamics, even when two different groups of animals were compared, and one group was measured in 2-bottle setup and the other in a 4-bottle design. We conclude that agreement of the parameters for the ADE group, and the different parameters for the no-ADE animals, not only confirm the usefulness of the new water-penalized net EtOH intake measure but also validate our ADE classification procedure.

Another advantage of the new measure is that we could compare ethanol consumption of animals receiving quinine-containing alcohol solutions during the AD phase to ethanol consumption of animals accessing quinine-free solutions. Because of the aversive taste the animals reduce consumption if quinine is added. Despite this, they achieve intoxication by increasing the alcohol/water ratio. The water-penalized net EtOH intake (which is based on the alcohol/water ratio) therefore was very similar in both conditions. Thus, our method provided a means to automatically select those animals that present inflexible drinking pattern in presence and absence of quinine. Thereby the (automatically selected) threshold on the penalized intake was the same for both groups.

Further analysis showed that the alcohol deprivation effect was much stronger in later cycles of repeated deprivation phases, and this agrees very well with other studies (Spanagel et al. 1996). Indeed, it has been demonstrated that animals develop loss of control over drinking behavior after several deprivation cycles, which shows that alcohol dependence is subject to development throughout consecutive deprivation phases.

The proposed measure provided a base for the development of a classification procedure into presenting ADE or no-ADE for single individuals. Such classification of individual animals according to their behavior during relapse-like situation is useful for many behavioral studies. One possible application is the evaluation of anti-relapse drugs (Litten et al. 2012), where the proposed approach enables the identification of animals in which the drug was effective. This would eventually help to identify genetic or metabolic variants related to the responding/non-responding phenotypes. Another very interesting application requiring individual classification would be to correlate patterns of earlier drinking phases with the ADE classification (Villarín Pildaín 2012). On this base it is conceivable that a prediction of the development of alcoholism is possible based on early drinking behavior. And finally, correlations between drinking patterns during earlier phases and the ADE output would provide another means of detecting phenotypes of (non-) responsiveness to drug treatment.

## Conclusions

We have shown that by penalizing alcohol intake by the amount of simultaneously consumed water we obtain much more meaningful information than by standard measures, like net intake or preference. We therefore recommend using this measure in further studies, especially if several alcohol solutions are provided. The applicability of the ADE/non-ADE classification approach depends on the paradigm and the drinking behavior of the animals. The approach might be useful for other animal models, but it would be necessary to test if the assumptions underlying our method are fulfilled, or if modifications are required to achieve applicability.
